# Lessons from a training needs assessment to strengthen the capacity of routine immunization service providers in Nigeria

**DOI:** 10.1186/s12913-019-4514-2

**Published:** 2019-09-14

**Authors:** Linda Arogundade, Titilola Akinwumi, Shola Molemodile, Ebubechi Nwaononiwu, Joshua Ezika, Inuwa Yau, Chizoba Wonodi

**Affiliations:** 1 Direct Consulting & Logistics, No.15, Amazon Street, Off Alvan Ikoku Way, Maitama, FCT, Abuja, Nigeria; 2 National Primary Health Care Development Agency Plot 681/682, Port Harcourt Crescent, Off Gimbiya Street, Area 11, Garki, FCT, Abuja, Nigeria; 3 Johns Hopkins Bloomberg School of Public Health, Rangos Bldg, Suite 600 855 N. Wolfe Street, Baltimore, MD 21205 USA

**Keywords:** Training needs assessment, Health workers, Health workforce, Continuing Professional Development, Continuing education, Adult learning, On-the-job training, Capacity strengthening, In-service tutors, Expanded Programme on immunization, Reaching every Ward, Nigeria

## Abstract

**Background:**

Health workers (HWs) providing routine immunization (RI) services play a crucial role in influencing vaccine uptake, a key determinant of improved immunization coverage. Over the years, Training Needs Assessments (TNAs) have not been routinely utilized in Nigeria to determine unmet needs of health workers offering immunization services and what approaches should be adopted to meet their training needs. The objective was to assess the level of Expanded Program on Immunization (EPI) knowledge among RI service providers and tutors in pre-service institutions in three Nigerian states, to identify unfulfilled training needs and their implications. It also sought HWs perception on a pilot training approach, where tutors will be used for in-service training.

**Methods:**

TNA survey tools were designed to obtain knowledge-based information on the fundamental EPI concepts through key informant interviews and focus group discussions with 90 HWs and 27 pre-service tutors. Quantitative data was also obtained, hence utilizing a mixed method approach for the study.

**Results:**

In spite of several previous trainings, HWs knowledge on basic immunization concepts including Reaching Every Ward (REW) strategy was varied and suboptimal. 83% of the HWs could not differentiate between the live attenuated and killed vaccines. In addition, pre-service tutors knowledge of fundamental EPI concepts, as well as HW perception of the new training approach also varied across the states.

**Conclusion:**

TNAs are valuable in determining specific training approaches to improve HWs skills needed to implement strategies required to increase vaccine uptake. However, EPI managers must be mindful of contextual factors beyond training needs such as finance and security, that can affect HW performance.

**Electronic supplementary material:**

The online version of this article (10.1186/s12913-019-4514-2) contains supplementary material, which is available to authorized users.

## Background

The health workforce is one of the key pillars of a health system in both developing and developed countries [1]. The provision of an efficient health service delivery, including immunization is heavily reliant on a skilled workforce to deliver quality services [2].

In sub-Saharan Africa, the health workforce is inundated with challenges including poor funding for staff salaries, little to no budgetary allocation for capacity strengthening efforts, inefficient deployment and poor distribution of health workers [3]. Serious gaps exist in the Health WorkForce (HWF) in the African Region, for instance, the densities of the following are lower in the region compared to global averages; by 6 fold for pharmaceutical and psychiatrist personnel, 5.4 fold for physicians and midwives, 4.8 fold for dentists and 2.4 fold for nurses [4].

In the Nigerian healthcare system, there is a major human resource shortfall both in quantity and quality as the doctor and nurse per 1000 ratio is 0.4 and 1.6 respectively and 0.2 for Community Health Extension Workers (CHEWs) [1].

At the community level, CHEWs are an important healthcare worker cadre providing basic health services such as immunization and antenatal care especially in the context of filling human resource void in Nigeria. Other categories of CHEWS who also provide health services at the community level are Junior Community Health Extension Worker (JCHEW), Environmental Health Technician (EHT), Environmental Technical Assistant (ETA). However, health worker distribution in Nigeria remains inequitable with mass exodus to perceived ‘greener pastures’ and concentration of the rest in urban areas [2].

In addition to the low numbers and inequitable distribution, health worker training is also poorly organized [5]. Usually, after the required pre-service training is completed, there is no clear process for continuing medical education as capacity strengthening is often tied to disease-specific interventions or programmatic activities especially in community settings.

Focusing on the immunization program in Nigeria, the country accounts for a quarter of over 2 million global under-5 deaths due to vaccine-preventable diseases (VPDs) [6]. This is quite alarming considering the role immunization plays as a cost-saving public health strategy for reducing childhood mortality and morbidity globally [7]. In 2016, the National Immunization Coverage Survey and Multi-Indicator Custer Survey (NICS/MICS) showed that only 23% of all eligible children in Nigeria received all the antigens in the national immunization schedule [8]. To reduce the burden of VPDs, the overburdened workforce is saddled with the responsibility of providing quality immunization services to the vast populace. As such, optimal skill building for health workers is required to manage the immunization program effectively and improve immunization coverage.

GAVI, the global vaccine alliance, has supported Low and Middle-Income Countries (LMICs) like Nigeria since its establishment in 2000 with funding new and under-used vaccines and health system strengthening activities. However, by 2028 Nigeria will be fully responsible for its immunization system financing completing its ‘graduation’ from GAVI funding [9]. Some of the criteria that has been used to measure the readiness and ability of countries to be self-sustainable after graduation from GAVI include; a resilient health system with skilled health workers, appropriate immunization policies and ample financial resources to accommodate the cost of required vaccines, storage equipment and remuneration of RI service providers [10]. Consequently, there is a need for EPI managers and stakeholders to cost-effectively manage available resources to ensure efficiency in all EPI activities.

### Cascade method of training for immunization service providers in Nigeria

**Fig. 1:**
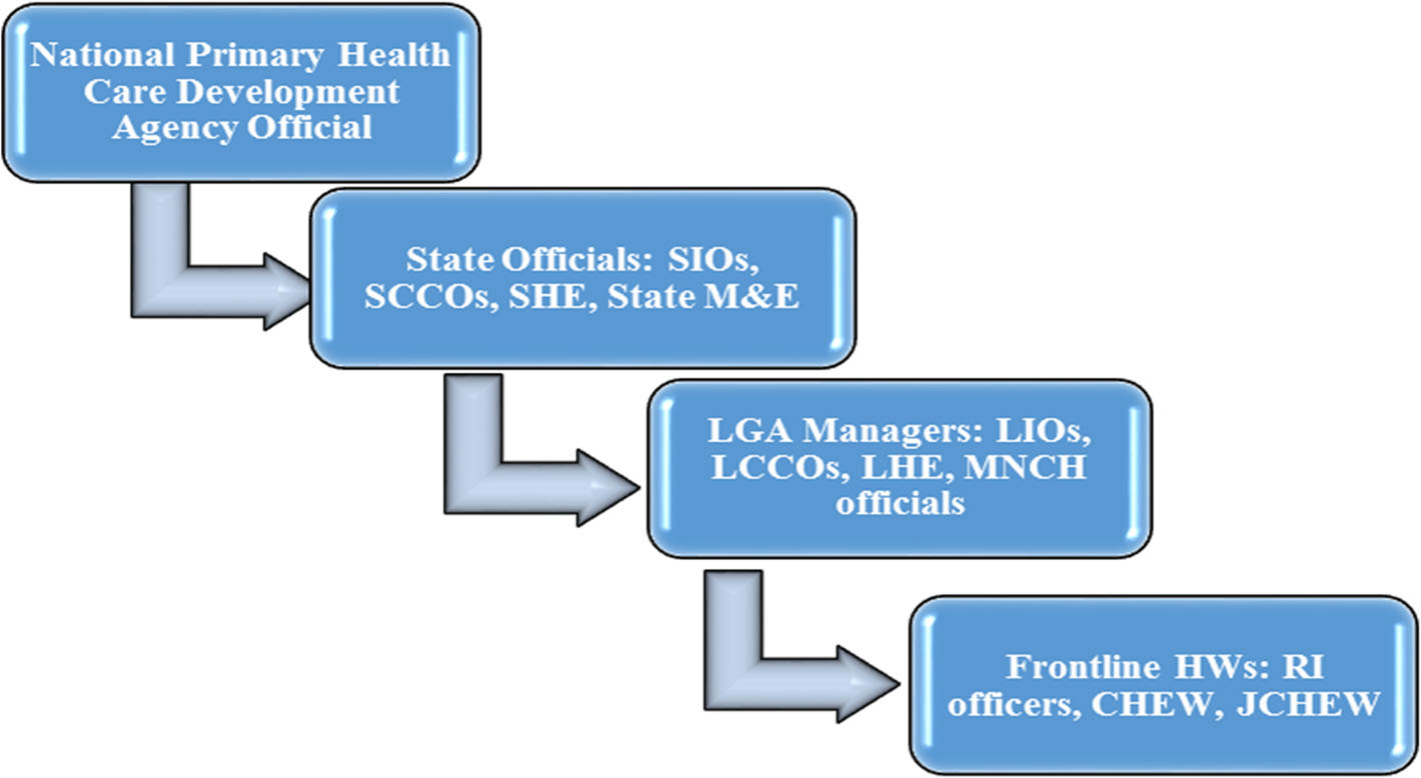
Current EPI training cascade in Nigeria

Nigeria, as with many LMICs, operates the cascade or ‘stream-lined’ Training of Trainers (ToT) approach to strengthen the capacity of routine immunization (RI) service providers [11]. This usually begins at the national level with training of national and select sub-national immunization program managers who cascade trainings to state officials. Thereafter, the state officials train the Local Immunization Officers (LIO) who finally train the frontline RI providers. To put more succinctly, the training is cascaded across 3 levels before it gets to the intended recipient – the frontline health worker (Fig. 1). However, based on the skill inadequacy of many RI providers, this training approach may not be as effective in meeting the needs of HWs. A likely explanation may be the absence of Training Needs Assessments (TNAs) to guide financial, technical and human resource allocation for HWs trainings [12].

TNAs play a vital role in identifying knowledge gaps, guiding how training content should be delivered and informing planning processes. There is little to no evidence to suggest that TNAs are routinely conducted in Nigeria before training of RI service providers in the PHCs [13]. Furthermore, there are no systematic approaches to determine the quality and knowledge of trainers at pre-service levels and EPI managers who conduct trainings to identify their own knowledge gaps and needs for effective training. For instance, significant knowledge gaps were found among tutors in pre-service training health institutions in two North-Western Nigerian states and HWs who passed through the institutions were not well equipped to deliver RI services [14].

To provide evidence on the value of TNAs to guide training of RI service providers, a TNA was conducted to identify knowledge gaps, training needs of RI service providers, and utlize ‘non-traditional’ tutors in health institutions in 3 states (Bauchi, Niger and Rivers) while also testing the feasibility of using these tutors drawn from tertiary institutions to provide training to HWs. This approach of using tutors from tertiary institutions to support EPI manager training proposes a shift from the norm of cascade training to an institutionalized system of training preceded by TNAs. HWs perception of this novel training approach was assessed including perceived effectiveness of previous cascade trainings.

## Methods

### Study sites

The study was conducted in 3 Nigerian states; Bauchi (Northeast), Niger (North-central) and Rivers (South-south). The selection of these states was purposive, based on the varied capacity and distribution of human resources for routine immunization across the different geographic zones in the country. A total of 11 local government areas (LGAs) were selected in the 3 states; 3 each in Niger and Rivers states and 5 in Bauchi state. LGAs were selected based on their RI performance and distribution by senatorial districts. A total of 12 pre-service training institutions in the 3 states were also engaged to identify and train tutors.

**Fig. 2:**
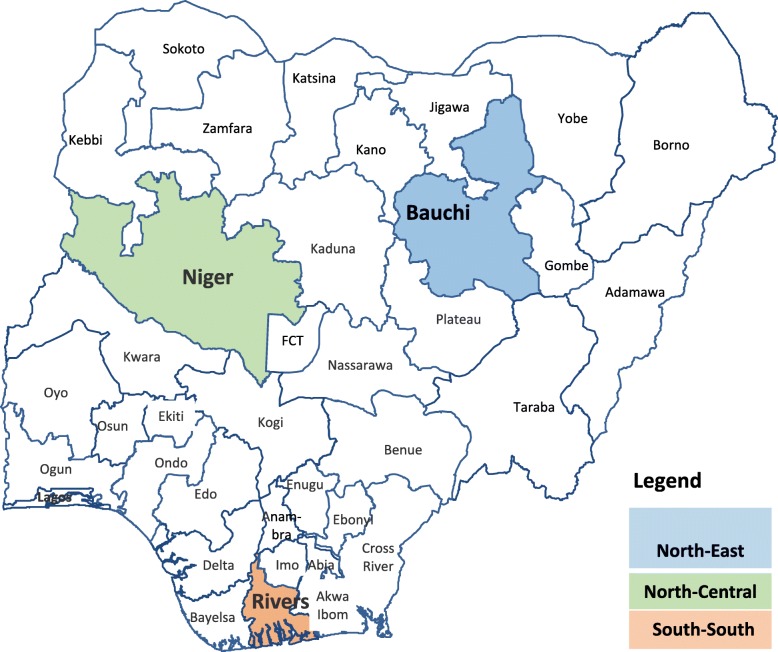
Nigerian Map showing study sites

**Source:** *National Population Commission. Image adapted from at* *www.population.gov.ng**. in August 2017* [15].

From the national population census conducted in 2006, Bauchi and Rivers states have an approximate population of 5 million each, while Niger has a population of approximately 4 million [15].(Fig. 2).

### Study population and sample size

A total of 117 health workers providing RI services and tutors serving in pre-service health training institutions in the three states were assessed; 90 health workers, mainly Community Health Officers (CHOs), Community Health Extension Workers (CHEWs) and Environmental Health Assistant/ Technicians (EHA/EHT) and 27 tutors from various pre-service training institutions, mainly from College of Medical Sciences, Schools of Health Technology, School of Nursing and Midwifery in the 3 states participated in the study (Table 1).

**Table 1:** Sample size distribution across states

State	HWs	Tutors
Bauchi	36	7
Niger	27	10
Rivers	27	10
Total	90	27

### Training needs assessment (TNA) tools

Structured data collection tools in survey format were specifically developed by the team for the TNA and administered to respondents who had provided consent to participate. The health worker tool was designed to obtain knowledge-based information about EPI with regards to the Reaching Every Ward (REW) strategy domains, general immunization concepts and previous EPI trainings they had attended in the last 5 years. The WHO REW strategy is aimed at improving immunization coverage in every ward (district) through the provision of quality and sustainable RI services [16]. The tool was also used to obtain key information on HW communication skills, particularly their ability to effectively communicate the *six key RI messages* to caregivers’ during immunization sessions. These six RI key messages are very important in enlightening caregivers about the vaccines, a number of visits needed to be fully immunized and the importance of keeping the immunization card safe. The six key RI messages are:

- Type of vaccines given
- Number of visits a child still needs in order to be fully immunized
- Adverse effects that may occur and how to treat them
- Place and time of the next immunization
- Bring the child back for immunization even if he/she is sick
- Taking good care of the immunization card and bringing it during the next visit

HW focus group discussions (FGDs) were also conducted to gain insight into previous trainings received as well as HW perception about the pilot training approach (Additional file 1). Informed consent was obtained from each participant with each FGD lasting about an hour and with 9 participants per FGD. At the end of each interview, recorded sessions which were collected with a tape recorder were transcribed and stored securely. Similarly, the tool for tutors was also structured in a survey format to obtain information on their tutoring experience, past EPI trainings attended and knowledge of EPI as described earlier (Additional file 2). Following development, the tools were pretested and refined before deployment.

### Data collection and analysis

The study team employed cross sectional study methods to obtain information from 117 participants across the 3 states using both qualitative and quantitative research methods. The qualitative data was obtained through In-Depth Interviews (IDIs) and Focus Group Discussions (FGDs) with RI officers, LGA managers and tutors from the different training institutions to elicit information on the ease of the process, acceptability, views, and barriers regarding the use of tutors to provide direct learning to HWs (Additional file 3).

Quantitative data was also obtained using structured questions to determine the number of HWs and tutors who ticked ‘yes’ on having knowledge of key EPI thematic areas such as REW strategy and basic immunization concepts on the 6 REW domains. The proportion was derived using ‘Count if’ for respondents who ticked ‘yes’ on the data entered in Microsoft Excel® and a mode determined for the tutoring experience and past EPI trainings attended.

Approval to administer tools was sought from the leadership of the PHC authority for health workers and health institutions for tutors in all the states. A tape recorder was used to record interviews to ensure that all responses were captured, aid the process of data analysis and improve retest reliability. Verbal consent was obtained from the participants before the commencement of the FGD. All responses to the questionnaires were collated and entered into MS-excel and cleaned. All the respondent’s information was analyzed using MS-Excel. Qualitative data was also transcribed and analyzed using thematic analysis to contextually describe and present emerging themes from interviews.

### Ethical approval

This was obtained from the National Health Research Committee (NHREC) in Abuja, Nigeria.

### Administrative approval

Study team also got approvals from the Johns Hopkins University International Vaccine Access Centre (JHU IVAC) in Baltimore USA, National Primary Health Care Development Agency, the State primary healthcare agencies, teaching institutions and PHCs in the 3 States.

## Results

Using the quantitative approach, the results were analyzed based on the level of knowledge per EPI thematic area while the qualitative analysis generated themes from the interviews conducted with the tutors and health workers. Themes from the qualitative interviews included Instructor competence, Training effectiveness, Experience in applying REW strategies, Training logistics & Perception of new training approach (using tutors for in-service trainings). Themes from the FGD are presented and described with supporting quotes from participants.

### Findings from health workers’ assessment

In Bauchi and Niger states, the majority of HWs providing RI services were males 31(83%) and 20(74%) respectively, while most in Rivers were females 24(89%). More than half of the respondents from Niger 14(52%) and Rivers 14(52%) were Community Health Extension Workers (CHEWs) while majority from Bauchi 22(61%) were Environment Health Technicians (EHT) and Environmental Health Assistants (EHA). Respondents had varied immunization work experience but overall over 80% had over one-year experience providing immunization services (Table 2).

**Table 2:** Bio-demographic characteristics of health workers across states

Characteristics of respondents	Bauchi n(%)	Niger n(%)	Rivers n(%)
Gender			
Male	31 (83)	20 (74)	3 (11)
Female	5 (17)	7 (26)	24 (89)
Present Designation			
Nurse	0 (0)	1 (4)	0 (0)
CHO	3 (8)	2 (7)	3 (11)
CHEW	8 (22)	15 (56)	14 (52)
JCHEW	3 (8)	6 (22)	8 (30)
Others (EHT/EHAs)	22 (61)	3 (11)	2 (7)
Years of immunization experience			
Less than 1	7 (19)	2 (7)	2 (7)
1–4 years	12 (33)	5 (19)	4 (15)
5–9 years	10 (28)	7 (26)	11 (41)
10–20 years	5 (14)	13 (48)	10 (37)

All respondents had attended one form of EPI in-service training on various EPI thematic areas including basic immunization concepts, REW, data management, cold chain management, Adverse Events Following Immunization (AEFIs), surveillance and injection safety in the last 5 year.

**Fig. 3:**
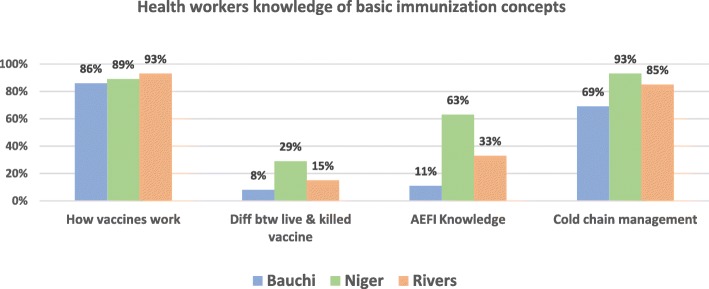
Health workers knowledge on basic immunization concepts

Generally, respondents across the three states had varied knowledge of basic immunization concepts. Their knowledge of the mechanism of how vaccines work and how cold chain management operates was sufficient across the states. However, respondents did not have sufficient knowledge on the difference between live attenuated and killed vaccines in the 3 states as over 50% could not tell the difference. Respondents in Bauchi and Rivers also didn’t have optimal (< 50%) knowledge of AEFIs (Fig. 3).

**Fig. 4:**
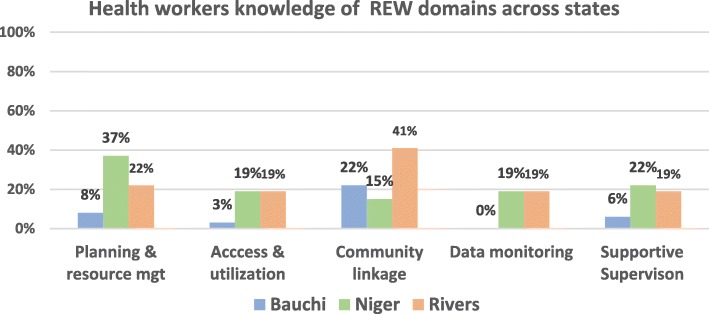
HWs knowledge on basic immunization concepts

Respondent’s knowledge of the five REW strategy domains for EPI was also varied and suboptimal in the three states as less than 50% of respondents in each of the states had knowledge of all five domains. The 5 EPI REW domains are; planning and resources management, access and utilization, community linkage, data monitoring for action and supportive supervision. Respondents in Bauchi performed poorly with regards to performance in the data monitoring and access and utilization domains (Fig. 4).

### HW’s ability to calculate immunization dropout rate

The dropout rate is one of the key EPI indicators used in measuring RI performance. Calculating the dropout rate helps health workers to monitor the progress of RI coverage. Most respondents in Rivers 22(81%) could perform this calculation while about two-thirds of the respondents in Bauchi 21(58%) and Niger 16(59%) were unable to. On average, only 53% of the respondents could successfully calculate drop out rates.

### HW’s proficiency in communicating the six key RI messages to caregivers

Health worker’competency level in communicating the six key RI messages was classified as ‘very effective’ if they could communicate all six, ‘moderately effective’ if they could communicate 3–4 messages and ‘not effective’ if they were able to communicate less than 2 messages.

In general, over half of all the respondents in the 3 states were moderately effective at communicating the key RI messages meaning that they could only communicate between 3 and 4 messages out of six. Respondents from Bauchi state had the highest 13(36%) proportion of those that were very effective at communicating the 6 key RI messages while Rivers state had the lowest 2(7%) (Table 3).

**Table 3:** Health workers proficiency level in communicating the six key RI messages to caregivers

State	Ability to communicate the 6 RI messages	Number(%)
Bauchi	Very effective	13 (36)
	Moderately effective	20 (56)
	Not effective	2 (6)
Niger	Very effective	5 (19)
	Moderately effective	16 (59)
	Not effective	5 (19)
Rivers	Very effective	2 (7)
	Moderately effective	17 (63)
	Not effective	8 (30)

### Findings from HWs FGD

Apart from the objective of establishing important knowledge gaps and training needs among health workers, this TNA also assessed RI providers’ perceptions about factors facilitating or limiting the application of Reaching Every Ward (REW) strategies. Some key findings from the FGD analysis are summarized under five major themes in Table 4.

**Table 4:** Findings from FGDs to assess previous trainings and perception of the new training approach

Theme	Key findings from FGD analysis
Instructor competence	• Majority expressed satisfaction with the approach employed by facilitator in previous trainings *“I like the teaching methods, the facilitators were friendly and we were taught how to plot the graph (in data tools management) and it improved our knowledge”*
Training effectiveness	• Some consented that previous trainings attended met up with their training expectations • Others felt the training should be improved upon by inclusion of practical and immediate demonstration. *“It helps to improve quality of the services especially by using the participatory methods of teaching. If they trained us by making the participants to do the reading or use the participants to demonstrate, it helps”*
Experience in applying REW strategies	• Varied preferences of the five REW domains. • Most HWs preferred the community linkage domain but experience difficulty in its application due to major logistic issues such as lack of funding for transportation, and refreshment for community stakeholders. • Insecurity and lack of funding hinder some of them from carrying out proper outreach *“The most difficult one to apply is Community linkage because getting the stakeholder in the community* i.e. *the Chiefs, the ward chairman is difficult because of their busy schedule”.*
Training logistics	• All the HWs experienced some training logistic challenges such as: ○ unconducive learning environment ○ inadequate training manuals ○ lack of financial incentives ○ poor refreshments ○ late notification for trainings *“We need posters and related IEC materials”* *“The logistics (especially money and manpower) are not okay and need to be improved”*
Perception of new training approach (using tutors for in-service trainings)	• Perceptions differed – ○ Some felt it was a good idea and believed using tutors can make a difference. ○ Others preferred the status quo because they doubted the ability of the tutors to do a good job since they have not been in the field for a long time. ○ Some gave the condition that tutors must be trained to update their knowledge on the new developments in EPI before they embark *“These teachers are only in the class and not in the field and our training needs practical skills. They should be trained before training us”*

### Findings from tutor’ assessments

With regards gender variation, all respondents in Bauchi were males while in Rivers state, majority 9(90%) were females (Table 4). In the last 5 years, all respondents had attended at least one training on key EPI domains. Respondents in Rivers state had attended more trainings than those from the other two states. Tutors from Niger state had more years of tutoring experience as half 5(50%) of them had been in service between 10 and 20 years, followed by tutors’ from Bauchi 3(43%) and Rivers 3(30%) who had also been in service for the same period. All others had tutoring experience between 1 and 9 years. The highest educational qualification among the tutors was a Ph.D. and the least, a Higher National Diploma (HND) degree (Table 5).

**Table 5:** Bio-demographic characteristics of tutors in the three states

Characteristics of tutors	Bauchi n(%)	Niger n(%)	Rivers n(%)
Gender			
Male	7 (100)	5 (50)	1 (10)
Female	0 (0)	5 (50)	9 (90)
Highest qualification			
PHD	0 (0)	0 (0)	2 (20)
Masters	3 (43)	3 (30)	2 (20)
BSc	4 (57)	6 (60)	5 (50)
HND	0 (0)	0 (0)	1 (10)
Tutoring experience			
Less than 1	0 (0)	0 (0)	0 (0)
1–4 years	2 (29)	2 (20)	4 (40)
5–9 years	2 (29)	3 (30)	3 (30)
10–20 years	3 (43)	5 (50)	3 (30)

**Fig. 5:**
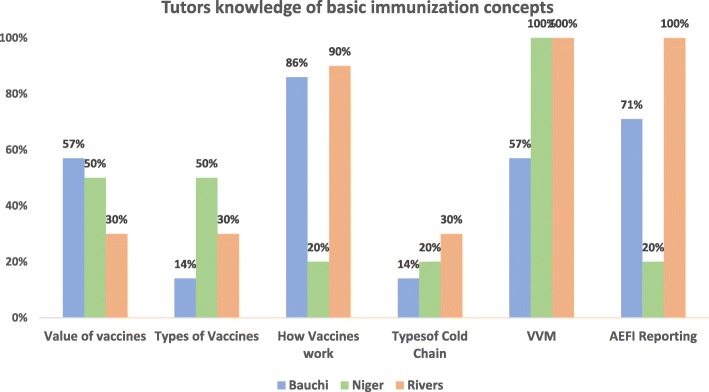
Tutors knowledge of basic immunization concepts

Generally, the tutors’ knowledge of the different basic immunization thematic areas and the five REW strategy domains varied across the three states. Tutors in Rivers state were generally more knowledgeable about basic immunization concepts such as vaccine mechanism of action, vaccine vial monitors and AEFIs compared to tutors from Bauchi and Niger (Fig. 5).

**Fig. 6:**
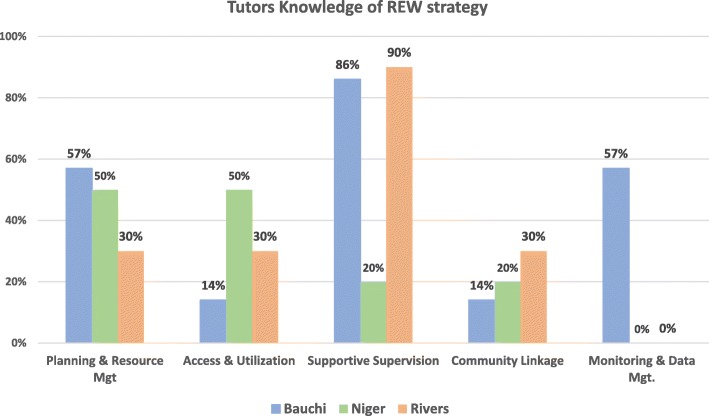
Tutors knowledge on REW strategy

There was also variability in knowledge of the five REW domains across states. However, major gaps were identified in the data monitoring for action and community linkage domains. Tutors from Niger and Rivers had no knowledge of the data monitoring for action domain while less than half of all the tutors in the three states had knowledge of the community linkage domain (Fig. 6).

## Discussion

One of the main drivers of high immunization coverage in many developing countries is the availability of sufficient and highly skilled health personnel who can effectively deliver available vaccines to children in and outside the health facility during outreaches [17].

Consequently, having an effective health system with skilled routine immunization (RI) service providers to deliver and manage these vaccines is as important as having the vaccine itself in order to attain the desired immunization coverage. Whilst the need to have a strong and viable health workforce delivering RI cannot be overemphasized; training of health workers is an essential component of all EPI activities, as it is capable of strengthening the quality of immunization programs in developed and developing countries [16]. The quality of training is also of paramount importance as it affects both the performance of HWs and the health outcomes of the country as a whole. In the last decade, countries like Pakistan have engaged effectively in training programs under several projects to bring about health system reform [17].

This TNA revealed that the knowledge and skills gap among some service providers and their inability to effectively deliver immunization services goes beyond just the lack of regular and well-structured training to improve knowledge. Instead, it reveals other deep-seated issues affecting primary health care delivery such as inadequate funding, frequent delays in the release of funds to carry out RI program activities, inadequate staffing mainly due to non-replacement of retired staff and other vital accountability issues in the system. Furthermore, many of the findings are valuable in informing the design and planning of training required to develop health worker skills with the resultant impact on routine immunization service delivery in Nigeria.

### Ensuring budgetary allocation for health worker trainings

It is vital for relevant financial authorities such as the Ministry of Finance and Health to prioritize training and other related health capacity strengthening activities into annual budgets and ensure timely and sufficient release for trainings in the country. Investing in capacity strengthening efforts for RI service providers will eventually translate to improved immunization coverage as health workers will become better equipped to implement strategies required to improve immunization coverage [18]. We found that critical elements of demand creation strategies for routine immunization uptake, such as community mobilization by health workers, were not well conducted because service providers were demotivated to carry out this activity. This was largely due to lack of funds for transportation and necessary logistics to mobilize caregivers and link RI services with the community. It has been established that when health workers develop a strong partnership with the community, immunization coverage tends to improve as a result of some level of trust developed that will allow the community to be more willing to accept and utilize services [17].

Improved RI coverage requires a multipronged approach that incorporates major drivers of enhanced service delivery including allocation and timely release of funds and having a highly skilled workforce to carry out community mobilization and deliver vaccines efficiently [4]. This is validated by this assessment, as timely release of funds to support health worker training and other sensitization activities in the community were identified as motivating factors for health workers to become more committed to ramping up efforts to improve immunization coverage.

### Training curriculum, content, and structure

The sub-optimal performance observed among health workers and tutors in this study with regards to basic knowledge and understanding of some core immunization concepts, points to major gaps in training structure and curriculum. This TNA revealed that even though majority of the tutors and health workers had attended more than one update training on RI within 1 year, their level of knowledge of basic RI concepts was poor. We also found that many of the HWs and tutors assessed, lacked the ability to determine immunization dropout rates, a fundamental requirement to assess how well the EPI program is performing. This may imply that there is an urgent need to restructure the trainings to address knowledge gaps identified. Routine immunization service providers stand to benefit from continuous learning on EPI if the training curriculum and content are structured to adopt a competency-based learning approach. This learning approach will help service providers easily understand core immunization concepts and allow them to develop concrete and fundamentals skills required for running a successful EPI program. Core principles of adult learning should also be considered when restructuring the training curriculum. This will help ensure that a significant portion of the time allocated for the trainings will focus on engaging them to do case demonstrations and other practical on-site activities as well as strengthen their capacity to use data to independently make decisions to improve the EPI program. The inability of service providers to determine basic but important indicators such as immunization dropout rates can derail the EPI program since this data is needed for action to address low coverage and improve immunization uptake.

Similarly, competency gaps were also identified in health worker ability to communicate the key immunization information required for caregivers. The ability of HW’s to effectively convey important RI information to clients is key to ensuring that caregivers bring their wards to complete the full immunization schedule and is invariably a determinant for immunization coverage. A study conducted in the Netherlands, highlighted information about vaccines as a factor that influences vaccination decision-making by parents who are keen on understanding how effective vaccines are, risks are associated with vaccination and adverse reactions [19]. Restructuring both the pre-service and in-service curriculum to address these gaps and other knowledge lapses highlighted in our study results will help reverse the present narrative and ultimately improve the quality of health personnel managing the country’s EPI program.

Even though opinions about the proposed method of using tutors in pre-service institutions to carry out in-service training varied among stakeholders assessed (Table 4), it is important to note that focus on improving the quality of trainings is the goal irrespective of training methods adopted. TNAs are key to improving the quality of trainings. Due to the dynamic and constantly evolving nature of EPI operations, there is a need for regular revision of the pre-service training curriculum to avoid any vacuum or disconnect between health training institutions and current EPI programmatic realities [20]. Both pre-service and in-service curricula should have clearly outlined learning objectives capable of addressing knowledge and skill gaps in vaccination theory and practice.

### Organization of trainings

Having an ideal environment for training sessions is a prerequisite to having a successful and impactful training. Ideal here means, appropriate tutor- trainee ratio (30:1) [21], conducive learning environment, clean conveniences, adequate supply of training materials, availability of Information Education and Communication (IEC) materials, light refreshments and decent accommodation [22]. In addition, we found that provision of stipends to cover travel expenses serves as a critical motivating factor [23].

The assessment revealed that service providers didn’t seem satisfied with the coordination of trainings in previous trainings attended. For instance, there were complaints about the lack of training manuals and IEC materials. Others noted that notifications for trainings outside their base were sent out very late and as a result, they are not able to prepare adequately for such trips. Not giving sufficient notice to service providers prior to trainings will result in late arrival of participants and sometimes complete absence. Service providers need time to put their health facilities and household together before they embark on any trip to the training location. While it may not be easy to get an ideal training setting, efforts should be made to provide comfortable environments for learning. It is important to note that, training coordinators at all levels need to be more accountable and shift focus from the number of service providers trained to the quality of training conducted. Conducting trainings in little clusters or batches over a period of time is more likely to be impactful as compared to a situation where a large number of service providers are trained at the same time. A study conducted in North-Central Nigeria to assess the knowledge and implementation of REW strategy among RI service providers showed that less than half of the study population (39.7%) understood the different approaches of expanding access to RI services [24]. The authors stated that some form of on the job training may be required to further strengthen the competence of RI service providers on REW [25]. Similarly, we found that continuous education was important to service providers. Training coordinators can improve quality of learning by incorporating on-the-job training to further strengthen the skills of service providers.

It is therefore important that early and adequate preparations should be made to ensure that necessary training materials are available and sufficient.

### Training in security-compromised settings

Some service providers during FGDs highlighted the sad fact that security challenges such as kidnapping and insurgency in certain parts of the country has jeopardized their freedom and threatened the EPI program. Some of the respondents complained that adequate security is not provided for them whenever they embark on outreach activities to those security-compromised areas. As a result, they have been exposed to danger and psychological stress. With such a situation, it becomes impossible for them to carry out services even when they have received quality training. It is pertinent for responsible authorities to provide the necessary security and ensure that conditions of service are suitable in order to guarantee workers safety and commitment to deliver on their assignment.

### Implementing an accountability framework

Some of the challenges affecting primary health care and immunization service delivery highlighted by services providers in this assessment are as a result of weak accountability. The main elements of accountability referred to here are financial, performance and political accountability. One of the most visible challenges that has the most direct link to accountability in this study is that of timely release of funds to implement trainings and other critical RI activities capable of improving immunization coverage. EPI programs in the country are mostly operationalized at the local government level where service providers and managers need to pay for important activities that precede immunization activities at the clinic and during outreaches. With respect to performance accountability, it is critical for managers and decision makers to agree on performance targets that will guide rewards and sanctions for service providers.

Implementable accountability frameworks are a major requirement for donors supporting health system strengthening and other health service delivery activities in Nigeria. There is need to strengthen existing accountability mechanisms in the country’s health system for improved immunization service delivery, improved efficiency in the use of resources and transparency.

### Implications for policy

Key recommendations posed to stakeholders and policy makers involved in training health workers providing routine immunization include but are not limited to the following; conducting TNAs before major trainings to ensure that training and module contents are targeted towards the needs of service providers; a revision of training curricula on all REW domains to make it more appealing for learners especially the data monitoring and access & utilization domains is imperative. Tutors and facilitators for EPI trainings require continuous learning with adult learning methods before they conduct any training. Evaluations of all trainings ought to be conducted using standard and nationally recognized tools to ascertain their immediate outcome and long-term impact.

Training curricula should be revised and reviewed at regular intervals to incorporate new information. Also, a restructuring of trainings to emphasize more practical hands-on approaches by implementing the 70:20:10 learning model [26]. Trainees should also be allowed to evaluate their tutors in order to rate their performance and identify areas that need improvement. Properly guided planning on trainings conducted by and for the institutions is important. Technical Working Groups (TWGs) and training units at each level (national, state and local) ought to be established or revitalized to coordinate trainings in the state. Implementation of Routine Immunization Supportive Supervision (RISS) should be continuously carried out considering that it is an important avenue for supervisors to interact with HWs and provide on-the-job mentoring and capacity strengthening activities.

### Study strengths and limitations

This study is unique, given that it examined the training needs of both RI service providers and tutors concurrently to demonstrate that poor performance of RI service providers might be in part linked to unmet training needs by tutors and facilitators. At the same time, the assessment proposed a paradigm shift from the 3-level cascade model to explore the use of pre- and in-service tutors drawn from health institutions to train RI providers directly.

The relatively small sample size and recruitment of health workers and tutors from the same geographical region may have excluded participants with potentially more varied perceptions on EPI, REW and immunization concepts. Also, while the study was conducted to test the feasibility of using tutors drawn directly from health institutions to train health workers, the approach was perceived as a challenging task to major stakeholders required to obtain buy-in and potential scale-up. As such, the proposal to reduce the number of cascade levels may have been seen as trying to obliterate the status quo, requiring much more stakeholder enagagement and consensus.

## Conclusion

This training needs assessment (TNA) identified knowledge and skills gap among RI service providers and tutors across major EPI thematic areas. The study also demonstrates that conducting TNAs is an important prerequisite for effective training because of the value of exposing not just knowledge gaps but other unmet training needs among service providers and facilitators. While the main objective of this TNA was to assess the current level of EPI knowledge among service providers and tutors, it also provided an opportunity to highlight other health system and service delivery issues affecting the EPI program such as timely release of salaries, allowances and other funds for immunization logistics. Our analysis indicates that weak accountability at different levels is responsible for many of the issues service providers encounter. While conducting regular update trainings or continuous learning for service providers and tutors is capable of improving their knowledge, it is important to restructure the training curriculum to a more structured competency-based model, improve training logistics, strengthen accountability and make deliberate plans for on-the-job training. These will potentially have a huge impact in improving the quality of trainings conducted. Finally, this assessment highlights that conducting TNAs has potential value for policy makers and program managers to guide planning and determining human resource needs.

## Supplementary informations

Additional file 1: Health Workers training tool. An instrument used for information-gathering on tutors years of experience, type of training(s) attended in the past, knowledge on EPI thematic areas. (PDF 931 kb)

Additional file 2: Tutors training tool. An instrument used for information-gathering on tutors years of experience, type of training(s) attended in the past, knowledge on EPI thematic areas (PDF 1793 kb)

Additional file 3: FGD for Health Workers & Tutors. A semi-structured interview guide assessing health workers and tutors perception about the novel approach of using in-service tutors for training. (DOCX 19 kb)

## Data Availability

Please contact the corresponding author with any queries as regards the availability of datasets supporting the findings of this study.

## Abbreviations

CHEW: Community health extension workers
CHO: Community Health Officers
EPI: Expanded Program on Immunization
FCT: Federal Capital Territory
NPHCDA: National Primary Health Care Development Agency
REW: Reaching Every Ward
RI: Routine Immunization
TNA: Training Needs Assessment
