# Supplementary material for: Lessons from a training needs assessment to strengthen the capacity of routine immunization service providers in Nigeria
Source: BMC Health Serv Res. 2019 Sep 14;19:664. doi: 10.1186/s12913-019-4514-2 (PMC6744655; doi:10.1186/s12913-019-4514-2)
Supplement: Supplementary file 1 — Health Workers training tool. An instrument used for information-gathering on tutors years of experience, type of training(s) attended in the past, knowledge on EPI thematic areas. (PDF 931 kb) [file 12913_2019_4514_MOESM1_ESM.pdf]

Strengthening Training for EPI & PHC in Nigeria (STEP-IN)
Training Needs Assessment Tool for Training Institutions Lecturers/Tutors

ENTRY INFORMATION	
Date: ____/_____/_______	Start Time of Questionnaire:
Interviewer Name	
Respondents Name	
Respondent's Current designation	
Respondents Phone Number	
Training Institution Name	
Address	
State	

Section A. General info (tick as appropriate)

1.	Age in years		
2.	Sex	Male	Female
3.	Teaching position	Assistant lecturers	
		Lecturer I	
		Lecturer II	
		Senior Lecturers	
		Professors	
		PHC Tutor 1	
		PHC Tutor 2	
		Lecturer	
		Others (specify)	
4.	Highest level of qualification	CHO	
		NCE	
		Degree (specify)	
		MSC(specify)	
		PhD(specify)	
		Fellowship	
5.	Type of training institution	University	
		Polytechnic	

		College of education	
		School of health technology	
		School of Nursing/Midwifery	
		Others	
6.	Number of years in (teaching) service	Less than 1	
		1-5	
		5-10	
		10-20	
		20+	

SECTION B. Expanded Program on Immunization related training

1.	Have you received any EPI training in the last 5 years?		☐ Yes	☐ No
2.	If yes who organized the trainings and when			
3.	Have you received any of these specific EPI training in the last 5 years?			Duration (Days Or Weeks)
i.	Vaccinology	☐ Yes	☐ No	
ii.	REW Strategy	☐ Yes	☐ No	
iii.	Vaccine/ cold chain Management	☐ Yes	☐ No	
iv.	New Vaccine Introduction	☐ Yes	☐ No	
v.	AEFI and Injection Safety	☐ Yes	☐ No	
vi.	Immunization surveillance training	☐ Yes	☐ No	
vii.	Immunization data management	☐ Yes	☐ No	

Section C. Vaccinology and Immunization basics

1.	Have you received any training on the basic principles of vaccines and the immune system?	☐ Yes	☐ No
	If yes, specify training topics (Yes/No)<br>Value of vaccines<br>Vaccine preventable diseases<br>Types of vaccines<br>How vaccines work<br>Others (specify)		

Section D. Reach Every Ward (REW) Strategy

1.	Do you have knowledge of REW Strategy?	☐ Yes	☐ No
I.	Planning and resource management	☐ Yes	☐ No
ii.	Improving Access and Utilization of Immunization Service	☐ Yes	☐ No
iii.	Supportive Supervision	☐ Yes	☐ No
iv.	Linking Services with Community	☐ Yes	☐ No
v.	Monitoring/ data management for Action	☐ Yes	☐ No
vi.	Others (specify/list)		

Section E. Vaccines and Cold Chain Management

1.	Do you have knowledge on the following?	☐ Yes	☐ No
I.	Vaccine vial monitor	☐ Yes	☐ No
ii.	Temperature monitoring	☐ Yes	☐ No
iii.	Vaccine storage conditions	☐ Yes	☐ No
iv.	Types of cold chain and cold chain maintenances	☐ Yes	☐ No
v.	Others (specify/list)		

Section F. Adverse Event Following Immunization (AEFI)

1.	Do you have any knowledge about AEFI?	☐ Yes	☐ No
I.	Types of AEFI (Mild and Severe)	☐ Yes	☐ No
ii.	Reporting	☐ Yes	☐ No
iii.	Referral and feedback	☐ Yes	☐ No
iv.	Injection safety	☐ Yes	☐ No

Section G. Client Relationship Management / Communication Skills

1.	Do you train your students on communication and interpersonal skills?	☐ Yes	☐ No
2.	If yes where is the training done	In school	☐
		In service (On the job)	☐
		External training	☐

Section H. Time availability for EPI training

1.	What is your current teaching schedule (Indicate actual number of days/week occupied)?	Busy all through ☐	Busy for 3-4 days in a week ☐	Busy for just 1- 2 days in a week ☐	Busy daily but with limited hours (1-3hrs) ☐	Other
2.	How interested are you in participating in STEP-IN trainings as a tutor	Not interested ☐	Little interest ☐		Very Interested ☐	
3.	How can the STEP-IN training best fit into your current schedule/curriculum? Provide best suitable times (3-4 options/year by month and weeks) Eg September 1 week for 10 days					

Section I. Experience in adult learning approaches and teaching styles

1.	What types of teaching style do you engage your students? (tick)	Lecture format ☐	Small group discussion ☐	Role play ☐	Case study ☐	Team-based problem solving ☐
2.	Which have been most effective and why?					

Section J. Perception of training gaps

1.	What are the current gaps in training methods for adult education? List

Questionnaire End time:
