# Supplementary material for: Lessons from a training needs assessment to strengthen the capacity of routine immunization service providers in Nigeria
Source: BMC Health Serv Res. 2019 Sep 14;19:664. doi: 10.1186/s12913-019-4514-2 (PMC6744655; doi:10.1186/s12913-019-4514-2)
Supplement: Supplementary file 2 — Tutors training tool. An instrument used for information-gathering on tutors years of experience, type of training(s) attended in the past, knowledge on EPI thematic areas (PDF 1793 kb) [file 12913_2019_4514_MOESM2_ESM.pdf]

Strengthening Training for EPI & PHC in Nigeria (STEP-IN)
Health Workers Training Needs Assessment Tool

The script is in bold.
Directions for the interviewers are italicized.

Introduce yourself. A sample script is below, for your reference:

My name is ________________________ and I am working on a health worker training project supported by BMGF and implemented by NPHCDA, Johns Hopkins Bloomberg School of Public Health and Direct Consulting and Logistics (DCL). In this project, we are trying to understand how we can improve health worker trainings for EPI and PHC.

The interview will last approximately 1 hour, 15 minutes. Some questions will be knowledge-based, whereas other questions will ask you about your on-the-job experiences.

Please note that all your responses will remain confidential and will not be shared with anyone or be linked to you in anyway. Your participation in this survey is not an individual performance evaluation. We will only use the answers to understand ways the Government and partners can improve future health worker trainings. Please note: You can end the interview at any time or skip answering any questions you do not feel comfortable with or do not wish to answer for any reason.

Do I have your permission to begin this interview? ___ Yes ____ No

Let's begin.

Please request the respondent's basic background questions and complete the form below.

ENTRY INFORMATION	
Date: ____/_____/_______	Start Time of Questionnaire:
Interviewer Name	
Respondents current designation	
Facility Name	
Address	
Ward	
LGA	
State	

I am going to ask you some general questions to understand you background and experiences. Begin filling out Section A of the form.

SECTION A. GENERAL INFO (tick as appropriate)

1.	Age in years						
2.	Sex				Male ☐	Female ☐	
3.	Present Qualification	Nurse ☐	CHO ☐	CHEW ☐	JCHEW ☐	Others (specify)	
4.	Highest level of degree awarded	Certificate ☐	OND ☐	HND ☐	BSC ☐	Others (specify)	
5.	Type of training institution attended				Public ☐	Private ☐	
6.	Number of years in service		Less than 1 ☐	1-4 ☐	5-9 ☐	10-20 ☐	Over 20 ☐
7.	Number of years providing immunization service		Less than 1 ☐	1-4 ☐	5-9 ☐	10-20 ☐	Over 20 ☐

For Section B question 1, ask the responded if they have been trained. Circle Y for yes, and N for no. If they respond 'Yes' ask respondent the duration for each training.

If they respond with 'No' move to the next question.

SECTION B. EPI TRAININGS

	Have you received any of these EPI trainings in the last 5 years?			Year trained	Duration (Days)
	REW Training	Y ☐	N ☐		
	Vaccine/ cold chain Management	Y ☐	N ☐		
	New Vaccine Introduction	Y ☐	N ☐		
	AEFI and Injection Safety	Y ☐	N ☐		
	Immunization Surveillance training	Y ☐	N ☐		
	Basic Guide for Service Providers (BGSP)	Y ☐	N ☐		
	Immunization Data management training	Y ☐	N ☐		

SECTION B1. CLIENT RELATIONSHIP MANAGEMENT / COMMUNICATION SKILLS

		Not effective	Moderately effective	Very effective
1.	**How would you rate your ability to communicate and pass adequate information to your patients/client?**<br>*Probe:*<br>*Did you show respect and empathy to them*<br>*Did you ask them if they need further explanation*<br>***Tick one choice***			

SECTION B1. CLIENT RELATIONSHIP MANAGEMENT / COMMUNICATION SKILLS

2.	***Are you able to communicate effectively the 6 key RI messages***<br>*1. Date and time for next visit*<br>*2. Vaccine given and diseases protected*<br>*3. Side effects and what to do incase*<br>*4. Importance of card retention*<br>*5. Even when sick, child should be brought to clinic*<br>*6. No of visits child needed to complete vaccination*<br>***Tick one choice***	Very Well (All 6)	Moderately well (3-4)	Fairly able (<2)
3.	***Do your patients relate back the information correctly to you in relation to the key RI messages? Do they ask for further clarifications? Record response here.***			
4.	***Have you received any training on how to improve interpersonal skills and communication with parents?***	**Y**	**N**	
	If the response is NO, skip questions 5 and 6, and go directly to question 7			

5.	***Where did you receive this training?***<br>*Tick as they apply*	In school	In service (On the job)	Other, please list:		
6.	Method of training	Formal	Informal	Supportive supervision		
7.	How important is it to have a good and friendly attitude towards the patients?		Not Important ☐	Slightly Important ☐	Highly Important ☐	
8.	**What are your specific suggestions on improving communications capacity among health workser?**		Please list:			

SECTION B2. TRAINING FEEDBACK

1.	**What training method do you find most effective?**<br><br>***Ask them to rank from most to least effective***<br><br>***Rank: 1 is best; 3 is least***	Instructor-led sessions, e.g., theoretical lecture followed by question & answer discussion<br><br>Rank: _____	Peer-to-peer learning, e.g., small group discussions, team-based learning<br><br>Rank: _____	Practical learning through role-playing different scenarios and solving case studies<br><br>Rank: ____	
	**What qualities do you want to see in a facilitator?**<br><br>**Check all that appl**	Friendly/ Approachable ☐	Very Formal ☐	Strict ☐	Nonchalant ☐

3.	**Have you ever been asked to formally provide feedback after a training? If so, when and method of feedback ?**	

SECTION C. KNOWLEDGE OF REW STRATEGY DOMAINS

How familiar are you with the REW strategy components?
Ask them first how many are they?
Ask them to list them and briefy explain about them.
Then for each components listed, circle the category that best characterizes their 'familiarity'

Planning and Resource management	Very familiar ☐	Fairly familiar ☐	Not familiar ☐
Improving Access and Utlization of Immunization services	Very familiar ☐	Fairly familiar ☐	Not familiar ☐
Supportive Supervision	Very familiar ☐	Fairly familiar ☐	Not familiar ☐
Linking Services with Community	Very familiar ☐	Fairly familiar ☐	Not familiar ☐
Monitoring and use of data management for Action	Very familiar ☐	Fairly familiar ☐	Not familiar ☐

Section C1. Planning and Resource management

1.	**How important is micro planning?** *Tick one answer*	Not important ☐	Slightly important ☐	Very important ☐	
2.	**What are the elements of a good micro planning?** ***Ask them to list any elements of the micro-planning. Then based on their response, please tick all that apply.*** ***If the response is not on the list, please list under 'other'***	Cover all areas and ensure no area is left out ☐	Optimize and use available resources ☐	Distribute workload equally and uniformly ☐	Other – please list:
3.	**Have you drawn a micro plan map?** *Tick one answer.*	Once ☐	Many times ☐	Never ☐	

4.	**What are the steps in developing HF micro planning?** ***Ask them to list the steps in developing a HF micro-plan.***	Analysis of data	Define catchment area/develop map	Develop work plan(session plan/needs requirement	Prioritize and budget	Other – please list:

	Then based on their response, please tick all that apply.<br><br>If response is not on the list, please list under 'other				
5.	Who did you involve to participate in the micro planning<br><br>*Ask them to list first. Then based on their response, please tick all that apply.*<br><br>*If response is not on the list, please list under 'other'*	Only health facility staff<br><br>☐	Only village health committee<br><br>☐	Community members<br><br>☐	Other – please list:<br><br>☐
6.	**How do you determine target population**<br><br>*Ask them to list or answer first. Then based on their response, please tick all that apply.*<br><br>*If response is not on the list, please list under 'other'*	Assumption<br><br>☐	Census population data<br><br>☐	Walk through/survey<br><br>☐	Other – please list:<br><br>☐
7.	**How do you determine your vaccine needs?**<br><br>*Ask them to list or answer first. Then based on their response, please tick all that apply.*<br><br>*If response is not on the list, please list under 'other'*	Using a Target Population method<br><br>☐	Previous consumption<br><br>☐	Vaccination session<br><br>☐	Other – please list:<br><br>☐

8.	**What are the parametes involved in TP method of determining vaccine needs?**<br>*Ask them to list or answer first. Then based on their response, please tick all that apply.*<br><br>***If response is not on the list, please list under 'other'***	Target population<br><br>☐	Desired coverage<br><br>☐	Wastage Factor<br><br>☐	No of Doses<br><br>☐	All of the above<br><br>☐

Section C2. Improving Access and Utilization of Immunization Service (Reaching target population)

1.	**What are the different strategies for routine delivery of RI services?**<br><br>*Ask them to list or answer first. Then based on their response, please tick all that apply.*<br><br>*If response is not on the list, please list under 'other'*	Fixed<br><br>☐	Outreach sessions<br><br>☐	Mobile strategy<br><br>☐	All of the above<br><br>☐	Other -please list:
2.	**What strategy is used to deliver RI services in remote and hard-to-reach areas with no HF?**<br><br>*Ask them to list or answer first. Then based on their response, please tick all that apply.*<br><br>*If response is not on the list, please list under 'other'*	Fixed<br><br>☐	Outreach sessions<br><br>☐	Mobile strategy<br><br>☐	All of the above<br><br>☐	
3.	**In analyzing your data what are the key questions you need to ask or check? Tick as they apply**<br><br>*Ask them to list or answer first. Then based on their response, please tick all that apply.*<br><br>*If response is not on the list, please list under 'other'*	Whether set target is achieved or being achieved<br><br>☐	What the data tells you about physical access (Eg Penta1)<br><br>☐	Whether those that have initial access continue (Utilization) (Drop-out)<br><br>☐	If vaccination schedule are respected?<br><br>☐	Other - please list

Section C3. Linking Services with community

1.	**How often do you involve the community in service delivery? eg planning, implementation and monitoring**<br>*Tick one answer*	Never ☐	Not often ☐	Every time ☐
2.	**How often do you carry out community sensitization to create demand for immunization?**<br>*Tick one answer*	Never ☐	Not often ☐	Every time ☐
3.	**How many times have your received training on community partnering / mobilization skills in the last 1 year?**<br>*Tick one answer*	Never ☐	Not often ☐	Two or more ☐
4.	**How often do you give feedback information to community about coverage, utilization disease outbreak to ensure community satisfaction?**<br>*Tick one answer*	Never ☐	Not often ☐	Every time ☐
5.	**How often do you solicit community input to solve problems and encourage them to have a sense of ownership of their health service delivery?**<br>*Tick one answer*	Never ☐	Not often ☐	Every time ☐

Section C4. Monitoring and Use of data for Action

1.	**Which of the basic data/RI information do you use to make decision?**<br>*Ask them to list or answer first. Then based on their response, please tick all that apply.*<br>*If something is not on the list, please list under 'other'*	RI Coverage data (Monitoring chart) ☐	Campaign Data ☐	Dropout rate ☐	VPD Cases e.g measles ☐	Supply information ☐	Child Imm. Register ☐
		Other - please list					

2.	**How familiar are you with the coverage indicators and core indicators for "monitoring for action"?**<br><br>*Tick one answer Probe: State core indicators (Coverage, Drop-out rate, un- immunized etc)*	Not familiar (don't know any)<br>☐	Slightly (Know at least 1)<br>☐	Very familiar (Know>2)<br>☐	
3.	**How conversant are you with the calculation of drop-out rate?**<br><br>*Tick one answer*<br>*Probe: Pent1-Pent3/Pent1*100*	No<br>☐	Yes<br>☐		
4.	**How many review meetings do you conduct at facility level per month to discuss data, trends and monitoring for action?**<br>*Tick one answer*	None<br>☐	Once<br>☐	Twice<br>☐	More than Twice<br>☐
5.	**How many review meetings did you participate at the ward level per quarter to discuss data, trends and monitoring for action?**<br>*Tick one answer*	None<br>☐	Once<br>☐	Twice<br>☐	More than Twice<br>☐
6.	**How many trainings have you received in the last 2 years on the use of data monitoring for action?**<br>*Tick one answer*	None<br>☐	Once<br>☐	Twice<br>☐	More than Twice<br>☐
7.	**What Routine Immunization (RI) data tools are you familiar with?**<br><br>***The HW should list by names the tools they are familiar with and then tick appropriately***				

Child Immunization Register	
Immunization monitoring chart	
Tally sheet	
Health facility summary form	
Vaccine management tool (VM1A & VM1B)	
Child Immunization card	
AEFI form	
Temperature monitoring chart form	
**Total (sum up all the category they are familiar with)**	__ / 8

Section C5. Supportive Supervision (tick as appropriate)

1.	**Have you ever received any supportive supervision in the last one year?**<br>*Tick one answer*<br>*If ans is N, skip Q2-7 below*				Y ☐	N ☐
2.	**Number of Visit(s)**<br>*Tick one answer*	1 ☐	2 ☐	3 ☐	4 ☐	5 or more ☐
3.	**Level of supervision**<br>*For the selected column based on answer choice above, tick the levels conducted supportive supervision visits. Tick all that apply.*	National ☐<br>State ☐<br>LGA ☐	National ☐<br>State ☐<br>LGA ☐	National ☐<br>State ☐<br>LGA ☐	National ☐<br>State ☐<br>LGA ☐	National ☐<br>State ☐<br>LGA ☐
4.	**Materials Used**<br>*In the same column selected above, tick which materials were used. If none were used, tick 'no'*	Checklist ☐<br>Other ☐<br>No ☐	Checklist ☐<br>Other ☐<br>No ☐	Checklist ☐<br>Other ☐<br>No ☐	Checklist ☐<br>Other ☐<br>No ☐	Checklist ☐<br>Other ☐<br>No ☐
5.	**Was Feedback given after supervision?**<br>*In the same column selected above, tick answer as appropriate*	Yes ☐<br>No ☐	Yes ☐<br>No ☐	Yes ☐<br>No ☐	Yes ☐<br>No ☐	Yes ☐<br>No ☐
6.	**If Yes, what type of feedback was given?**<br>*In the same column selected above, tick answer as appropriate*	Oral ☐<br>Written ☐	Oral ☐<br>Written ☐	Oral ☐<br>Written ☐	Oral ☐<br>Written ☐	Oral ☐<br>Written ☐
7.	**How effective and useful was the supervision?**<br>*Tick one answer*	Not Effective ☐<br>Slightly Effective ☐<br>Very Effective ☐	Not Effective ☐<br>Slightly Effective ☐<br>Very Effective ☐	Not Effective ☐<br>Slightly Effective ☐<br>Very Effective ☐	Not Effective ☐<br>Slightly Effective ☐<br>Very Effective ☐	Not Effective ☐<br>Slightly Effective ☐<br>Very Effective ☐
8.	**In what ways, can supportive supervision help/assist you in improving the quality of services you deliver?**<br>*Please note their comments*					

SECTION D. BASIC VACCINOLOGY KNOWLEDGE (Tick as appropriate)

1.	**How do vaccines work?**<br><br>*Tick as appropriate. For other response, list in comment box*	By boosting Immunity ☐	Act as drug to treat infection ☐	Don't know ☐
		Other - please list		
2.	**What type of vaccine do you know ?**<br>**Give examples for each type.**<br><br>*Tick appropriate box based on the answer, then ask for an example for each type of vaccine they know.*	Live Attenuated ☐<br>List an example	Killed/In activated ☐<br>List an example	Toxoid ☐<br>List an example
3.	**Do you understand why Yellow Fever vaccine is given once and pentavalent is given three times?**<br><br>*Ask for an explanation for each vaccine. If they are able to explain that yellow fever vaccine provides lifetime immunity AND that penta requires repeated dosage to confer immunity, check 'well understood.'*<br><br>*If they can only provide one out of the two explanations, check 'somewhat understood'*<br><br>*If they cannot explain, check 'does not know'= Killed vaccine (require repeated dose to confer immunity)*	Well understood ☐	Somewhat understood ☐	Does not know ☐
4.	**Are you familiar with part or all the EPI schedule?**<br><br>*Tick one answer*		Y ☐	N ☐
5.	**Do you understand the current EPI schedule by time, age and frequency**<br><br>*If they answer both of the questions correctly (answers below), mark 'well understood'. If they are able to answer only one question correctly, 'mark somewhat understood.'*	Well understood ☐	Somewhat understood ☐	Does not know ☐

	**If no answer is correct, mark 'does not know.'**<br><br>*1 How many times a child need to be at facility to be fully immunized? Answer:*<br><br>*2. What are the vaccines/antigens given at each contact? Answer:*			

SECTION E. VACCINE AND COLD CHAIN MANAGEMENT

1.	**What does Vaccine Vial Monitor (VVM) indicate?**<br><br>*Tick one answer*		Exposure level of vaccines to heat	Exposure level of vaccines to cold	
2.	**At which VVM stage will you discharge your vaccine?**<br><br>*Tick one answer*		Stage 2 and below?	Stage 3 and above	
3.	**What temperature do you maintain all your vaccines?**<br><br>*Tick one answer*	Room Temperature	Freezing	Between +2 to +8℃	Others specify
4.	**When do you measure temperature of your CCE?**<br><br>*Tick one answer*	Morning	Afternoon	Evening	
5.	**Does your facility use fridge tags?**<br><br>**If yes, ask:**<br>*Have you been able to use it correctly to read/monitor the temperature? Have you faced any problems using it?*<br><br>*Please record answer in comment*	Yes	No	Comment:	

SECTION F. ADVERSE EVENT FOLLOWING IMMUNIZATION (AEFI)

1.	**Do you know what AEFI stands for?**<br><br>**If answer is: Adverse Event Following Immunization, tick 'yes'**		Y ☐	N ☐
2.	**How knowledgeable are you about AEFI?<br>Tick one answer**	**Very knowledgeable**	**Fairly knowledgeable**	**Not knowledgeable**
3.	**List the types of AEFT that needs to be reported.**			

	Note: Respondent should list the AEFIs they know and tick the following based on the list below. If their response is not only the list, please list under other:					
	Injection site abscesses					
	BCG lymphadenitis					
	Immunization related hospitalization within one month of the shot					
	Unusual medical incident related to immunization eg continuous high body temperature, rashes etc(within one month of vaccination)					
	All deaths thought to be related to immunization					
	Other: Convulsion, Coma					
4.	Are you familiar with the AEFI standard reporting and investigation forms?<br>*Tick one answer*			Y ☐	N ☐	
5.	Do you have AEFI forms in your facility?			Y ☐	N ☐	
6.	If yes, how often do you use the report tool ? Tick one answer		Monthly reporting ☐	Case by case basis ☐	Others ☐	
7.	**Did you give feedback to the community after investigating the AEFI?**<br>***Tick one answer***			Y ☐	N ☐	
8.	**What method of feedback did you use?**<br>***Tick ALL that apply. If others, please list.***	written report ☐	Via circular report ☐	Community meeting ☐	Other, please list:	
9.	**Who was involved or included in the feedback?**<br>***Tick ALL that apply. If others, please list.***	Only facility staff ☐	LGA Staff ☐	Advocacy group ☐	Community leaders ☐	Others; please list

SECTION G. KNOWLEDGE OF BASIC PROGRAM MANAGEMENT IN EPI

1.	**Are you familiar with writing reports on activities performed**<br>***Tick one answer***<br>***Probe: Is report with a heading, dates, background, activities key findings, recommendations.***	Not familiar ☐	Slightly familiar ☐	Very familiar ☐
2.	**What method do you use to prepare reports?**<br>***Tick ALL that apply.***	Hand written ☐	Typing on computer ☐	Send via email ☐
3.	**Do you make your report available within the required timeline?**<br>***Tick one answer***	Never ☐	Occasionally ☐	Every time ☐

4.	**Do you organize regular meetings on how to resolve problems in the facility with other staff?**<br>*Tick one answer*	Never<br>☐	Once a while<br>☐	Regularly<br>☐
5.	**Are you familiar with how to develop work plans and timelines?**<br>*Tick one answer Probe: Does it have the objective, activities, responsible, output/outcome*	Not familiar<br>☐	Have an idea<br>☐	Very familiar<br>☐
6.	**Have you received any training on how to prepare work plans and write reports in the last 5 years?**<br>*Tick one answer*	Never<br>☐	Once<br>☐	Two or more<br>☐
7.	**How do you communicate with fellow workers? Supervisors?**<br>*Tick one answer*	Very formal<br>☐	very friendly and respectful manner<br>☐	Unfriendly and rude<br>☐

End of Questionnaire

Thank you so much for your time
