# Supplementary material for: Lessons from a training needs assessment to strengthen the capacity of routine immunization service providers in Nigeria
Source: BMC Health Serv Res. 2019 Sep 14;19:664. doi: 10.1186/s12913-019-4514-2 (PMC6744655; doi:10.1186/s12913-019-4514-2)
Supplement: Supplementary file 3 — FGD for Health Workers & Tutors. A semi-structured interview guide assessing health workers and tutors perception about the novel approach of using in-service tutors for training. (DOCX 19 kb) [file 12913_2019_4514_MOESM3_ESM.docx]

**FGD for States on how to improve quality of training for EPI managers**

***Focus Group Discussion Guide/protocol***

**Consent Process**

Participants can complete consent forms in advance once we know those participating or it can be completed on the day of the interview. Ensure that participants understand the information in the consent form.

**Draft consent form.**

*Thank you for agreeing to participate in the FGD. We are very interested to hear your valuable opinion on how we can improve the quality of EPI training for frontline health workers and EPI managers.*

***Objective of the FGD***

*The major objective of the FGD/study is to learn how satisfied you are with quality of EPI training you have been receiving so far and how we can improve the quality of these trainings by finding out which are the key immunization training issues you still have challenges/problemto help inform the way future trainings should take place. Are issues in some specific domains (eg AEFI, cold chain management, injection safety, data management etc) or are issues specific training methodology/style such as facilitation methods, training methods, training logistics (e.g. type of venue, feeding, transportation etc.), and ratio of trainers to trainees .Or could there be other issues outside these?*

- *The information you provide us is absolutely confidential, and we will not associate your name or the name of your facility with anything you say or reveal in the focus group.*
- *We would like to record the FGD so that we can make sure to capture the thoughts, suggestions, opinions, and ideas we hear from each member of the group. No names will be attached to the focus groups and the tapes will be destroyed as soon as they are transcribed.*
- *The discussion is voluntary and you may choose not to answer any question or withdraw from the study at any time.*
- *We understand how important it is that any information you provide here is kept private and confidential. We will ask each participant to respect each other’s confidentiality and opinions.*
- *If you have any questions now or after you have completed the FGD, you can always contact a study team member like me, or you can call the STEP-IN project team coordinator or manager whose names and phone numbers are on this form.*
- *Please kindly write your name and sign to show that you agree to participate in this focus group.*

*Name -----------------------*

*Signature-------------------*

*Date------------------------*

*STEP-IN Project team contact information.*

*National Project Coordinator- Pharm Inuwa Yau -08035982969*

**Conducting the FGD**

**Introduction:**

1. Welcome

Introduce yourself and any other person assisting you such as the note taker e.tc., and pass around the Sign-In Sheet with a few quick demographic questions (age, gender, cadre, no of years in service, no of years at this facility) to the group while you are introducing the participants of the focus group.

*Review the following:*

- Who we are and what we’re trying to do
- What will be done with this information
- Why we asked you to participate

1. Explanation of the process

Find out from the group if anyone has participated in a focus group before. Explain that focus groups are being used more and more often in health and human services research.

*About focus groups*

- We learn from you (positive and negative)
- We are not trying to achieve consensus, we’re gathering information
- In this project, we are doing both questionnaires and focus group discussions. The reason for using both of these tools is that we can get more in-depth information from a smaller group of people in focus groups. This allows us to understand the context behind the answers given in the written survey and helps us explore topics in more detail than we can do in a written survey.

*Logistics*

- Focus group will last about an hour and half
- Feel free to move around
- Where is the bathroom? Exit?
- Help yourself to refreshments

1. Ground Rules

Ask the group to suggest and come up with some ground rules. After they brainstorm some ground rules, make sure the following are on the list.

- Everyone should participate.
- One person speaks at a time
- There are no right or wrong answers
- Information provided in the focus group must be kept confidential
- Stay with the group and please don’t have side conversations
- Turn off cell phones if possible
- Everyone should relax and have fun

1. **Turn on Tape Recorder**
2. Ask the group if there are any questions before we get started, and address those questions.
3. Introductions

- Go around table:

*Begin discussion, move slowly and at their pace and ensure you give participants time to think before answering the questions. Use the probes to make sure that all issues are addressed, but move on when you feel you are starting to hear repetitive information.*

***Questions***

1. Let’s begin by discussing the type of EPI training you have had in the past, where, when, and how it was done?
   1. What did you liked about these trainings
   2. What did you not like about the training (issues/challenges experienced, observed and or perceived). Start the discussion on 1) the timing of the training, 2) method of the training, 3) how it was facilitated and attitude of the facilitator, 4) Logistics issues (venue, accommodation, feeding, allowance) 5) trainer/trainee ratio etc.). Ask if there are other issues??
2. Based on the training received so far, which of the REW domain training did you find most easy to understand and apply? Which ones were very difficult to understand and apply? What suggestion do you have on how to make the difficult REW components easier to understand and apply and what exactly will you want to be improved?
3. What is your view if teachers from health training institution are brought in to provide in-service training for EPI? What kind of incentives (e.g.- certificates, stipend, venue, feeding, facility recognition, and logistics etc.) do you consider will boost the quality and outcome of EPI training? Rank them in other of preference.
4. Should the quality of the training improve, do you think it will reflect in your performance and output? Do you think on the long run it will improve the immunization indicators and child hood mortality indices? What is your perspective?
5. What are you views about the use of Teachers to provide to you (at facility level) complementary supportive supervision and mentorship after training? Can it add any value to what you do at health facility level? Do you think it can work? What will you suggest?

***Some Probes for Discussion****: (more can be added)*

- *Training environment/atmosphere eg if its conducive, friendly, safe etc.*
- *Benefits/incentives ie stipend/perdiem, certificate of attendance*
- *Attitude of facilitators eg if they are friendly, give feedback, carries participants along, etc.*
- *Relationship with facilitators.*
